# Bimodal ankle-foot prosthesis for enhanced standing stability

**DOI:** 10.1371/journal.pone.0204512

**Published:** 2018-09-26

**Authors:** Sara R. Koehler-McNicholas, Billie C. Savvas Slater, Karl Koester, Eric A. Nickel, John E. Ferguson, Andrew H. Hansen

**Affiliations:** 1 Minneapolis Department of Veterans Affairs Health Care System, Minneapolis, MN, United States of America; 2 Division of Rehabilitation Science, Department of Rehabilitation Medicine, University of Minnesota, Minneapolis, MN, United States of America; University of Colorado Boulder, UNITED STATES

## Abstract

Previous work suggests that to restore postural stability for individuals with lower-limb amputation, ankle-foot prostheses should be designed with a flat effective rocker shape for standing. However, most commercially available ankle-foot prostheses are designed with a curved effective rocker shape for walking. To address the demands of both standing and walking, we designed a novel bimodal ankle-foot prosthesis that can accommodate both functional modes using a rigid foot plate and an ankle that can lock and unlock. The primary objective of this study was to determine if the bimodal ankle-foot system could improve various aspects of standing balance (static, dynamic, and functional) and mobility in a group of Veterans with lower-limb amputation (n = 18). Standing balance was assessed while subjects completed a series of tests on a NeuroCom Clinical Research System (NeuroCom, a Division of Natus, Clackamas, OR), including a Sensory Organization Test, a Limits of Stability Test, and a modified Motor Control Test. Few statistically significant differences were observed between the locked and unlocked ankle conditions while subjects completed these tests. However, in the absence of visual feedback, the locked bimodal ankle appeared to improve static balance in a group of experienced lower-limb prosthesis users whose PLUS-M mobility rating was higher than approximately 73% of the sample population used to develop the PLUS-M survey. Given the statistically significant increase in mean equilibrium scores between the unlocked and locked conditions (*p* = 0.004), future testing of this system should focus on new amputees and lower mobility users (e.g., Medicare Functional Classification Level K1 and K2 prosthesis users). Furthermore, commercial implementation of the bimodal ankle-foot system should include a robust control system that can automatically switch between modes based on the user’s activity.

## Introduction

Postural stability, defined as the ability to control the body center of mass (COM) within a given base of support, is essential to many activities of daily living [1]. Among healthy individuals, a combination of physiological systems (i.e., visual, vestibular, and somatosensory) are used to maintain postural stability. Together, these systems regulate motor control strategies used to limit body COM movements (i.e., static postural stability), voluntarily shift the body COM within the base of support (i.e., dynamic postural stability), and control the body COM in the presence of a perturbation (i.e., functional postural stability). When one or more of these physiological systems are compromised, the capacity for individuals to appropriately control their body COM is diminished. For example, numerous studies have reported that somatosensory losses associated with a lower-limb amputation often lead to poor balance and balance confidence [2–4], resulting in significant barriers to community participation, health outcomes, and quality of life. The capacity for lower-limb prostheses to restore postural stability is therefore an important aspect of rehabilitation among this population.

**Fig 1:**
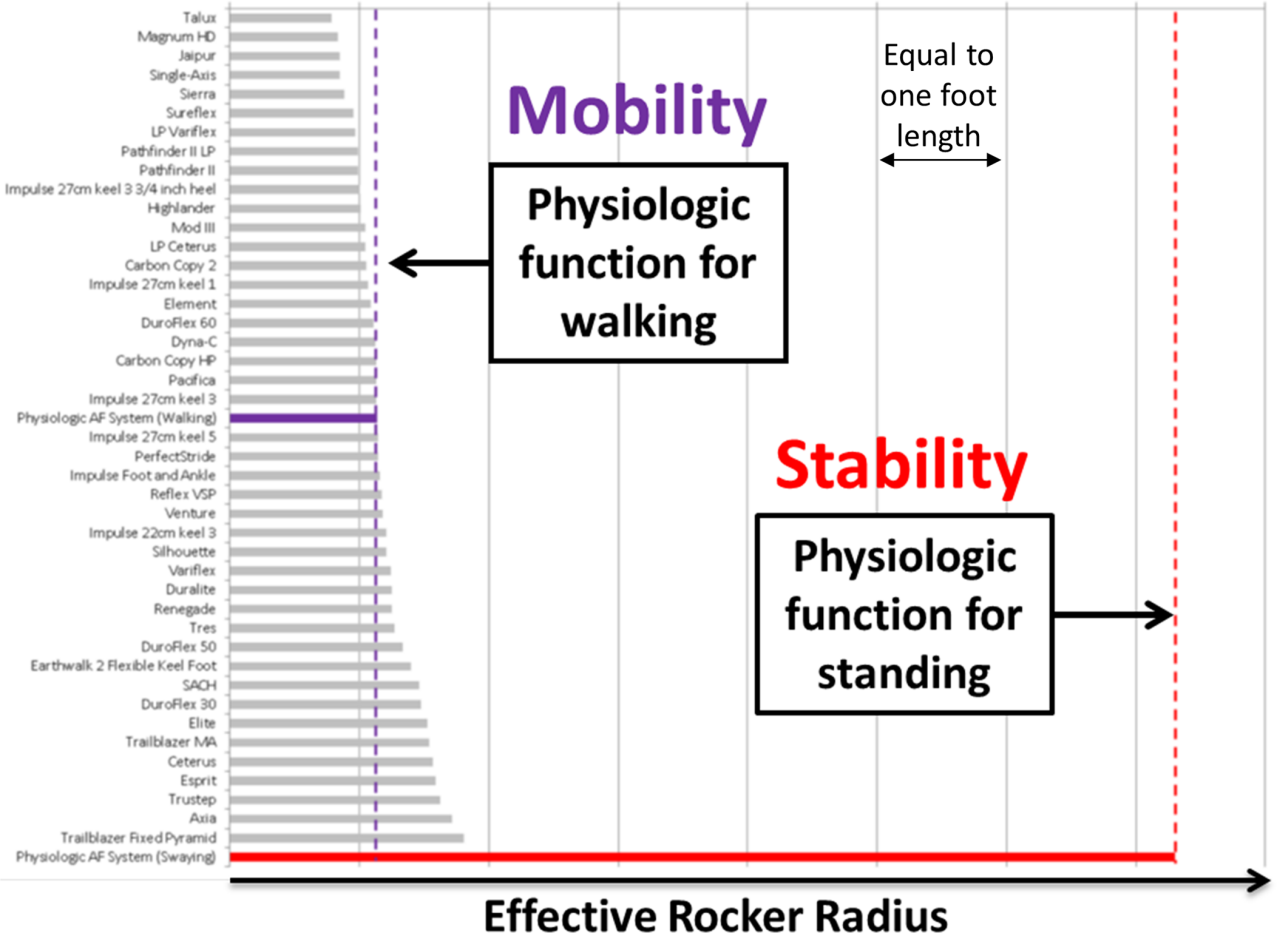
Radii (normalized by foot length) of effective rocker shapes of 44 commercially available mechanically passive prosthetic feet (gray), the anatomical ankle-foot (AF) system for walking (purple), and the physiologic AF system for fore-aft swaying (red). These results suggest that the focus of most current prosthetic foot designs is on walking and not standing and swaying.

Previous work suggests that in order to restore static and dynamic postural stability for individuals with lower-limb amputation, ankle-foot prostheses should be designed with a flat effective rocker shape for standing [5]. However, most commercially available ankle-foot prostheses are designed with a curved effective rocker shape for walking. Fig 1 illustrates this point, showing the effective rocker shape radii (normalized by foot length) for a representative sample of 44 commercially available mechanically passive prosthetic feet (circa 2008) compared to an anatomical ankle-foot system while standing/swaying and walking. To obtain these data, nine companies and organizations provided prosthetic feet for mechanical testing as described by Hansen et al. [6]. Rocker shapes for the anatomical ankle-foot system were obtained according to methods described by Hansen et al. [7]. Consistent with a previous study of effective rocker shapes [5], Fig 1 shows that the rocker radius of the anatomical ankle-foot system during standing/swaying is considerably larger than that of walking. More notably however, prosthesis radii data clustered around the physiologic function of walking highlights the focus of most current prosthetic ankle-foot designs to favor the demands of walking over standing.

Recent advances in ankle technology have begun to address this disparity, including a powered system described by Shultz et al. [8], which incorporates an algorithm to modulate the equilibrium angle of the ankle in order to adapt to ground slope and the user’s posture while standing. The extent to which this feature improves standing stability, however, has not been described. The development of a passive ankle-foot prototype (i.e., the Rock’N’Lock Foot) with separate modes for standing and walking has also been described by Adamczyk [9]. In the walking mode, this system provides a curved but rigid rocker shape for mobility. In the standing mode, this system provides a rigid arched base for stability. The Rock’N’Lock Foot is an interesting design that may provide good stability for standing. However, balance tests have not been conducted with prosthesis users to determine if the standing mode of the Rock’N’Lock improves standing function.

**Fig 2:**
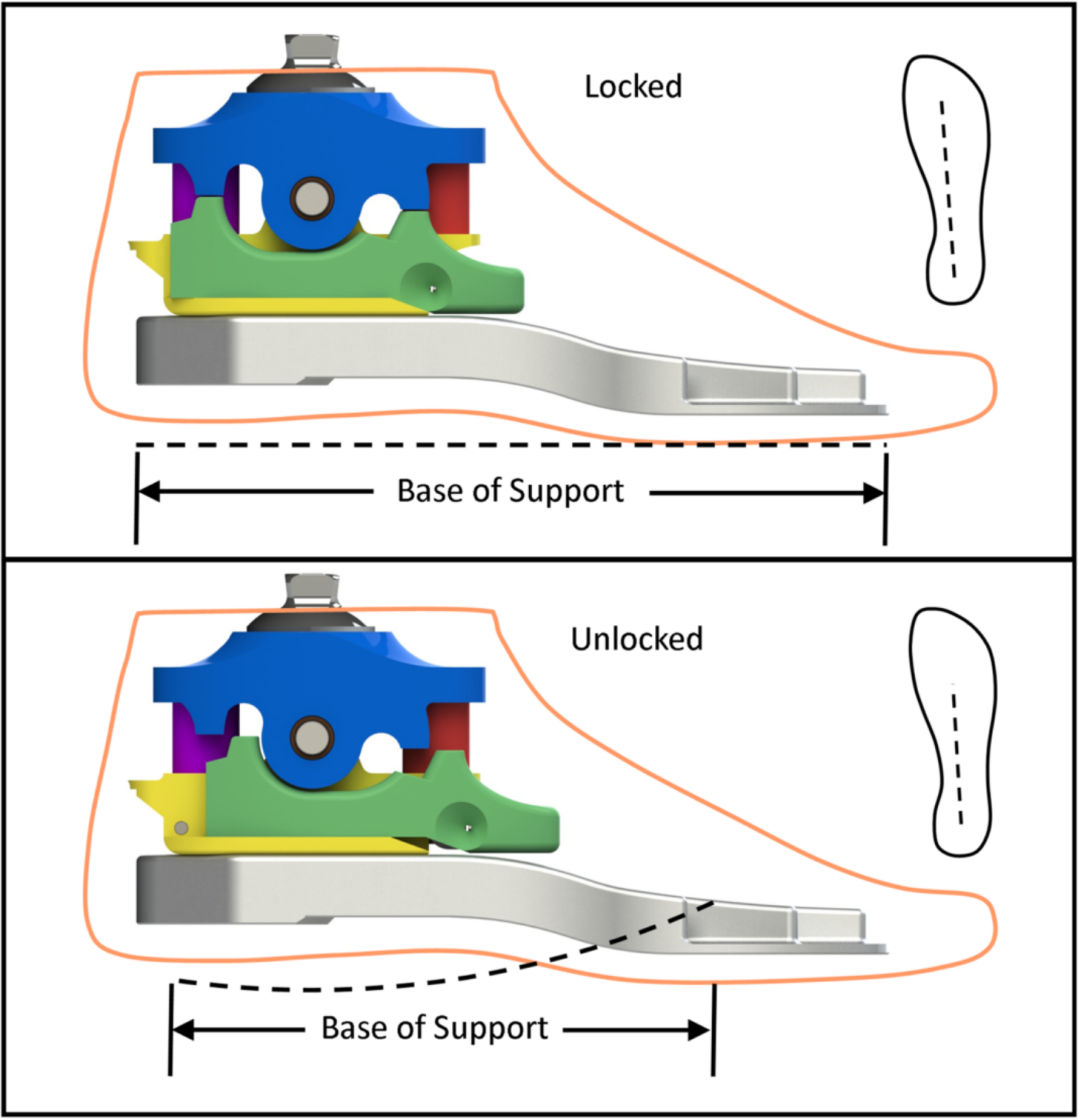
CAD renderings of the ankle-foot prosthesis in the unlocked mode (top) and locked mode (bottom). Mode switching is controlled by a slider (green) that is pushed and pulled by an actuator (not shown). The durometer of rubber bumpers (red and purple) located on the anterior and posterior portions of the ankle block are selected according to the weight and activity level of the user.

To address the demands of both standing and walking, we designed a novel bimodal ankle-foot prosthesis [10] that can accommodate both functional modes through the use of a rigid foot plate and an ankle that can lock (resulting in a flat effective rocker shape for standing, Fig 2, top) and unlock (resulting in a curved effective rocker shape for walking, Fig 2, bottom). To switch between these modes, the ankle-foot system incorporates a small linear actuator (Firgelli L-12; Firgelli Automations, Ferndale, WA) that pushes and pulls a slider (Fig 2, green). In the standing mode, the slider is in a position that mechanically blocks ankle motion. Consequently, the base of support of the ankle-foot system is equal to the length of the rigid foot plate, approximating the shape of the anatomical ankle-foot system during standing. In the walking mode, the slider is in a position that allows ankle motion, which is governed by the durometer of two rubber bumpers located on the anterior and posterior portions of the ankle block (Fig 2, purple and red). With the ankle free to rotate, the base of support of the ankle-foot system is reduced compared to the standing mode. However, compression of the rubber bumpers results in a curved rocker shape that can be optimized to match that of the anatomical ankle-foot system during walking.

Given the potential for this system to address current limitations in passive prosthesis technology, the primary objective of this study was to determine if the bimodal ankle-foot system could improve various aspects of standing balance and mobility in Veterans with a lower-limb amputation. With regard to static balance, we hypothesized that subjects would exhibit higher equilibrium scores during quiet standing tasks when the ankle was locked compared to when it was unlocked, and that this effect would be exaggerated in the absence of visual feedback. With regard to dynamic balance, we hypothesized that subjects would increase their functional base of support during voluntary leaning tasks when the ankle was locked compared to when it was unlocked, and that the largest effect would be observed during leaning tasks toward the prosthesis. Finally, with regard to functional balance, we hypothesized that subjects would be able to tolerate perturbations of the standing surface with fewer balance failures when the ankle was locked compared to when it was unlocked, particularly when the contribution of the sound limb to recover from the perturbation was limited. We also investigated the effect of the bimodal ankle on walking mobility and hypothesized that the unlocked bimodal ankle would not diminish mobility outcomes compared to the subjects’ usual prosthesis. Collectively, these hypotheses were designed to explore whether future commercial implementation of the bimodal ankle-foot system could lead to improved standing balance and balance confidence among lower-limb amputees, improving participation in social activities and quality of life.

## Methods

### Recruitment

This study was approved by the Institutional Review Board Subcommittee, the Subcommittee on Research Safety, and the Research and Development Committee of the Minneapolis VA Health Care System (MVAHCS): 4494-A Bimodal Prosthetic Ankle-Foot System for Improved Balance and Mobility. A convenience sample of Veterans with unilateral transtibial amputation receiving treatment at the MVAHCS were recruited for this study according to the following inclusion criteria: ≥18 years old, at least six months post-discharge from inpatient rehabilitation with a prosthesis, body mass less than 125 kg, able to understand the document for informed consent, and able to perform multiple tests of balance and mobility. Unilateral prosthesis users who had disabilities affecting their contralateral limb were considered eligible for study participation. Veterans with visual impairment, skin breakdown on their residual limb, or a poor prosthetic socket fit that reduced their ability to control the prosthesis were excluded from the study. Prior to enrollment, all subjects signed a consent form approved by the MVAHCS Institutional Review Board. Following this consent process, a certified prosthetist inspected the skin integrity of the subject’s residuum and evaluated their prosthetic socket fit.

### Experimental protocol

All testing occurred during a single study visit. Following the informed consent process, a structured interview was conducted to collect subject demographics. Subjects then completed the Activities-Specific Balance Confidence (ABC) scale [11] and the Prosthetic Limb Users Survey of Mobility (PLUS-M) to describe the use of their usual prosthesis. The ABC scale is a self-efficacy measure of confidence in performing various mobility related tasks without falling or experiencing unsteadiness. It has been validated for use among individuals with lower-limb amputations [12] and has been shown to correlate with the 2-Minute Walk Test and the Timed “Up & Go” Test [2]. The PLUS-M, a 12-item tool developed using modern psychometric methodology (i.e., Item Response Theory), has been recommended for use in both clinical and research settings as a brief, reliable, and precise measure of lower-limb prosthesis user’s mobility [13–16].

Subjects then practiced three balance tests (see following section) using a NeuroCom Clinical Research System (NeuroCom, a Division of Natus, Clackamas, OR). During this testing, subjects wore their usual prosthesis (Table 1) and their usual shoes. Subjects practiced each test at least once, and were encouraged to repeat testing until they felt comfortable with the apparatus and understood how to properly perform the test. They also completed two functional mobility tasks with their usual prosthesis, including a 10-meter walk test (10MWT) and an L-Test. The 10MWT measures gait speed over a 10-meter walkway and is a common outcome measure used to track rehabilitation progress. In older adults, a difference of 0.13 m/s has been defined as a substantial meaningful change in gait speed [17]. The L-Test is a performance measure of walking ability designed for individuals with lower-limb amputation [18]. It is a 20-m test of basic mobility skills that includes two transfers and four turns. Subjects completed both the 10MWT and the L-Test at their normal walking speed and repeated each test three times.

**Table 1:** Perturbations that were used for the modified Motor Control Test (MCT).

Perturbation Size	Amplitude of Perturbation	Duration of Perturbation (ms)
Small	0.006944 * height	250
Medium	0.017361 * height	300
Large	0.031250 * height	400

The amplitude of perturbation (both forward and backward) was scaled to the patient’s height (NeuroCom Clinical Operations Guide, a Division of Natus, Clackamas, OR) and presented in a random order.

A certified prosthetist then disconnected the subjects’ usual prosthesis from their socket by loosening two adjacent screws on the pyramid connector to preserve alignment. The bimodal ankle-foot system was attached to the subjects’ socket using an appropriately sized pylon and was fitted to the subject per the following clinical procedure: 1) bench alignment with the bimodal ankle locked, 2) static alignment with the bimodal ankle locked, and 3) dynamic alignment with the bimodal ankle unlocked. Bumpers were selected according to the weight of each subject in order to achieve an appropriate roll-over shape during walking [5] then adjusted to suit individual subject preference. Subjects wore their same (usual) shoes and were blinded to the design features of the bimodal ankle, which was concealed by a spectra sock, prosthetic foot shell, and outer sock. During the alignment process, subjects walked with the unlocked ankle first in a set of parallel bars and then through a series of hallways. When the prosthetist was satisfied with the alignment of the prosthesis and the subject was comfortable, subjects repeated the 10MWT and the L-Test with the bimodal ankle in the unlocked mode.

Subjects were then asked to remove their prosthesis, which was taken to an adjacent room and set to either the locked or unlocked mode by a member of the study team (testing order was randomized and the investigator collecting the data was blinded to the testing condition). Balance testing was repeated with this first testing condition. A video record of the testing protocol was obtained with two GoPro HERO 4 cameras (GoPro, San Mateo, CA), which were used to document any balance failures during testing. After completing the first testing condition, the prosthesis was removed, the ankle was set to the remaining condition, and balance testing was repeated.

At the conclusion of the study, the function of the bimodal ankle was revealed to the subject and a semi-structured interview was used to collect the subjects’ impressions of the ankle-foot system. Subjects were also asked to provide suggestions for future design iterations.

### Balance testing

For all balance testing, subjects were secured with an overhead safety harness. The harness was adjusted to keep subjects a safe distance from the surrounding structure of the NeuroCom in the event of a balance failure. However, care was taken to ensure that the safety harness did not interfere with the subjects’ ability to perform the following balance tests:

#### Sensory Organization Test

To assess static postural stability, all subjects performed the first two conditions of the Sensory Organization Test (SOT) on the NeuroCom Clinical Research System. The SOT has been designed to identify abnormalities in a patient’s use of three sensory systems that contribute to postural stability (i.e., visual, vestibular, and somatosensory). The first two conditions of the SOT have been designed to evaluate the contribution of the somatosensory system. During testing, subjects were instructed to stand quietly for three, 20-second trials, first with their eyes open and then with their eyes closed. Subjects stood with their feet shoulder width apart and with their arms at their sides. Force plates continuously measured the center of pressure under each foot, which was used to calculate an equilibrium score (i.e., the difference between the theoretical range of normal antero-posterior sway (12.5°) and the maximum range of sway of the subject). Equilibrium scores were calculated in NeuroCom’s software as a percentage based on degree of sway from vertical, with a higher score indicating less sway.

#### Limits of Stability Test

**Fig 3:**
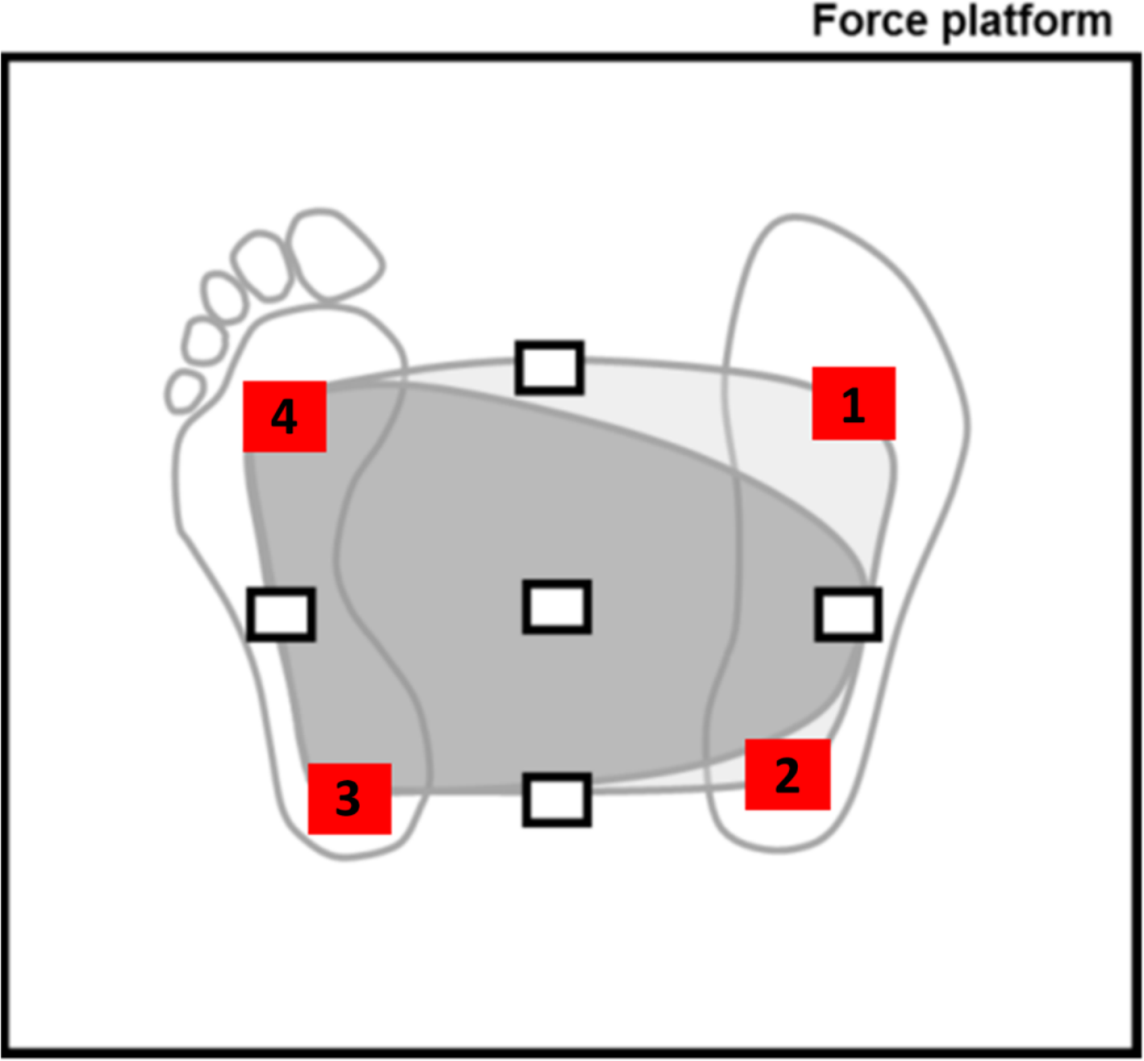
The Limits of Stability (LOS) test involves voluntary movements of the subject’s center of mass (COM) within their functional base of support. The theoretical functional base of support of an able-bodied population is shown in light gray. The dark gray (asymmetric) region represents the theoretical base of support of a unilateral amputee population, with a smaller base of support on the prosthetic (right) side. The rectangles show targets for the standard LOS test. In this study, subjects were instructed to lean toward the four diagonal targets (labeled 1–4).

To assess dynamic postural stability, subjects also completed a Limits of Stability (LOS) test on the NeuroCom Clinical Research System. The LOS has been designed to quantify the maximum distance a patient can intentionally displace their COM in four cardinal and four diagonal directions while maintaining stability. Previous studies have shown that the functional base of support of individuals with lower-limb amputation tends to be smaller on the prosthetic side compared to the sound side (Fig 3), suggesting that the sound side plays a key role in maintaining balance following amputation [19,20]. To explore the contribution of the bimodal ankle to dynamic postural stability, this study used an abbreviated version of the LOS test, which focused on the ability of subjects to displace their COM in four diagonal directions. Unlike the cardinal directions directly in front of and behind the subject’s COM, which necessitate balanced weight distribution on both forefeet or heels (respectively) and thus do not isolate the weight distribution on a single forefoot or heel, the diagonal directions investigated in this study specifically challenged the forefoot and heel of each foot while minimizing the contribution of the contralateral foot. During testing, subjects were given visual feedback about their COM (represented on a computer monitor) and were asked to voluntarily shift their COM toward a target, the location of which was determined using reference data collected from a group of able-bodied subjects (see Fig 3 for identification of targets 1–4). At the beginning of the test, subjects were instructed to maintain their COM within a center target and wait for a visual and auditory cue. Upon receiving the cue, subjects leaned in the direction of the target, attempting to place their COM as close as possible to the target. Target locations were normalized by the subjects’ height and subjects were given eight seconds to complete the task. At the end of the task, subjects were instructed to move their COM back to the center target and wait for the next visual cue. To quantify dynamic stability, the maximum excursion of the COM toward each target was analyzed. To analyze LOS results for the prosthetic forefoot, data from targets 1 and 4 were averaged for right- and left-side amputees, respectively. To analyze results for the prosthetic heel, data from targets 2 and 3 were averaged for right- and left-side amputees, respectively. For the purposes of presenting a graphic depiction of these results, LOS results were mirrored for left-side amputees so that the amputated side corresponded to the right side (i.e., targets 1 and 2).

#### Modified Motor Control Test

**Fig 4:**
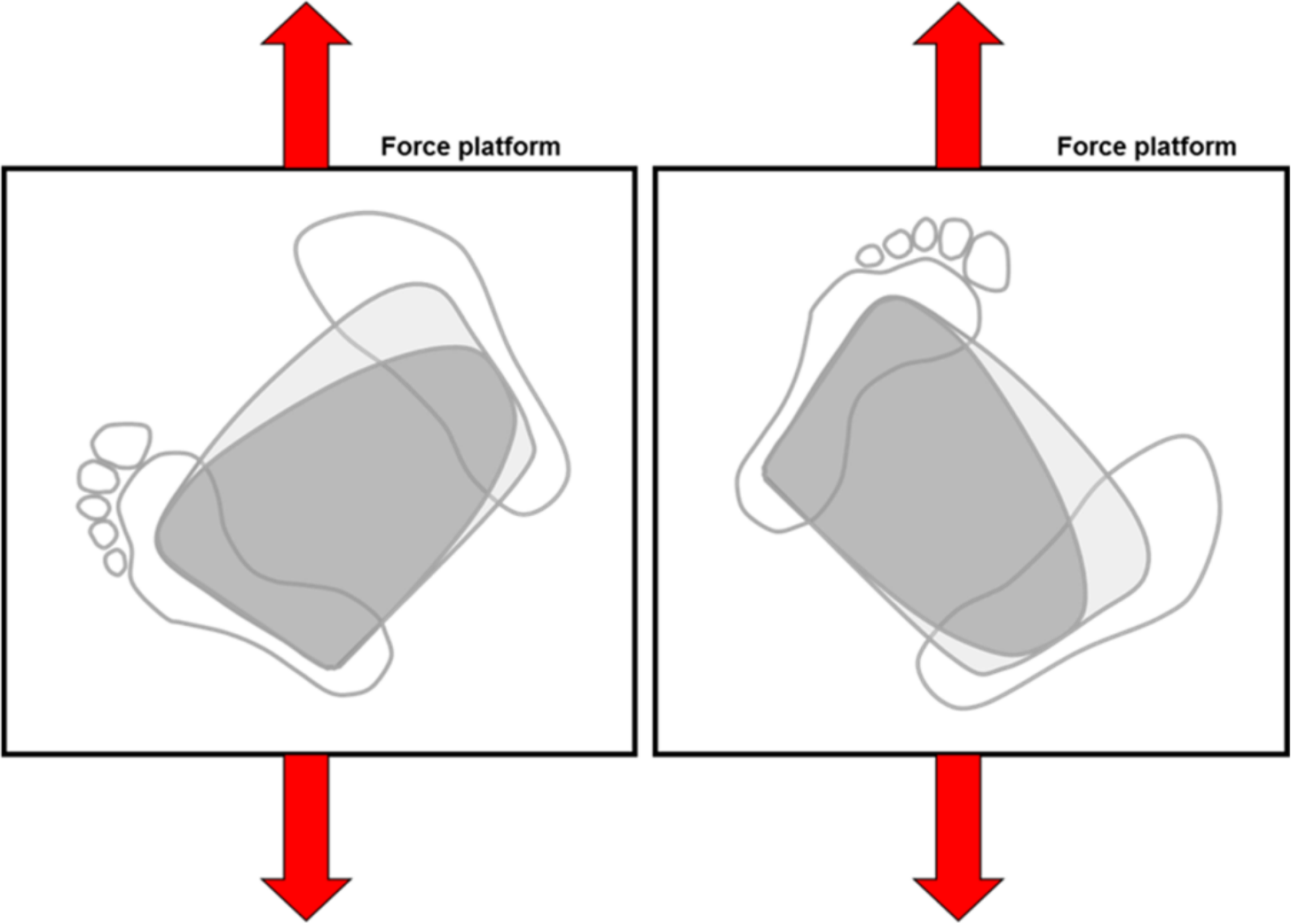
During the modified Motor Control Test (MCT), subjects were positioned at a 45° angle to the direction of force plate translation and random fore-aft perturbations were administered. In this depiction, the configuration on the left challenges the forefoot of the prosthetic side and the heel of the sound side, while the configuration on the right challenges the forefoot of the sound side and the heel of the prosthetic side.

To assess functional postural stability, subjects performed a modified version of the motor control test (MCT). The MCT is a standard balance protocol provided by the NeuroCom Clinical Research System that incorporates translational perturbations of the standing surface and measures the patient’s ability to quickly recover from an unexpected external disturbance. In the standard clinical test, sequences of small, medium, and large platform translations (three of each in sequential order) are delivered in the forward and backward direction, while subjects stand so that they are aligned with the direction of platform translation. Platform translations are scaled to the patient’s height according to the equations shown in Table 1. In this study, we modified the standard MCT for unilateral transtibial prosthesis users by instructing subjects to stand at a 45° angle on the force platform (Fig 4) and randomized the order (both amplitude and direction) of platform perturbations. This altered standing position reduced the ability for subjects to always rely on the sound limb, since off-angle perturbations shifted balance requirements to one forefoot or one heel (depending on the perturbation direction) rather than to the center of both forefeet or heels. The modification used in this study thereby isolated the performance of the forefoot and heel regions of the ankle-foot system. Balance failures, defined as a trial in which the subject took a step, reached out and touched the visual surround of the NeuroCom, or fell into the overhead harness, were documented and described in the subjects’ study record.

### Statistical analysis

All statistical analyses were performed using SPSS 19 for Windows (SPSS Inc., Chicago, IL). Parametric methods of statistical analyses were applied in this study after confirming all data followed a normal distribution. To compare SOT results across subjects, mean equilibrium scores were calculated for each subject and analyzed using a repeated measures analysis of variance (ANOVA) with two within-group factors (MODE: LOCKED versus UNLOCKED and EYES: OPEN versus CLOSED). To compare LOS results across subjects, a repeated measures ANOVA was performed on maximum COM excursion data with two within-group factors (MODE: LOCKED versus UNLOCKED and TARGET: 1–4). Diagnostic testing for this analysis included Mauchly’s test of sphericity. When sphericity was violated, a Greenhouse-Geisser correction was applied. In both repeated measures ANOVA analyses, post-hoc Bonferroni multiple comparisons were run as indicated by a significant interaction term (*p*<0.05). In SPSS, Bonferroni-corrected *p*-values were calculated by multiplying uncorrected *p*-values by the number of comparisons made. These corrected *p*-values were then compared to an alpha level of statistical significance set at *p*<0.05. To analyze the modified MCT results, the total number of balance failures across all trials was tallied; however, given the rarity of a balance failure in this study, no statistical analyses were applied to these data. Finally, a paired t-test was used to compare mean walking speed and L-test results between the unlocked bimodal ankle and the subjects’ usual prosthesis (*p*<0.05).

## Results

### Subject demographics

Data were collected from 1 female and 17 male Veterans with unilateral transtibial amputation (Table 2). Amputation etiology varied across subjects and included trauma (e.g., combat injury, motorcycle accident, farm accident), vascular disease (e.g., diabetes mellitus, Buerger’s disease), and cancer. The mean age, mass, and height of the group was 58 ± 14 years, 89 ± 14 kg, and 176 ± 6 cm, respectively. Subjects rated their balance confidence 82 ± 12 points on a 100-point scale (range: 56–96). For comparison, ABC scores below 67 points have been associated with an increased risk of falling in older adults [21]. The mean PLUS-M t-score across subjects was 55.8 ± 5.9 (range: 45.8–64.5), corresponding to a mobility rating that was higher than approximately 73% of the sample population used to develop the PLUS-M.

**Table 2:** Subject demographics.

ID	Gender	Age (yrs)	Height (cm)	Mass (kg)	TSA (yrs)	Amputation Etiology	Usual Prosthesis	ABC	PLUS-M t-score
1	M	44	180	58	3	Tumor	Ossur Re-flex Rotate	56	45.8
2	M	66	183	87	47	Combat	Freedom Agilix	82	53.6
3	M	31	173	76	10	Combat	Rush Dynamic Flex Foot	91	54.4
4	F	55	160	84	9	Motorcycle	Ossur Flex-Foot Axia	63	47.1
5	M	67	175	86	6	Tibiotalar Fracture	Freedom Renegade	91	57.3
6	M	66	180	78	4	Vascular	Freedom Highlander	81	52.7
7	M	76	176	90	12+	Vascular	College Park Venture	59	46.4
8	M	35	175	95	9	Combat	Ossur Re-flex Rotate	92	61.0
9	M	71	170	83	7	Vascular	Ossur Vari-flex	67	52.7
10	M	67	175	84	11	Farm accident	College Park Soleus	78	53.6
11	M	68	193	112	16	Cancer	College Park TruStep	94	62.5
12	M	53	180	117	8	Vascular	Ossur Vari-flex XC	84	54.4
13	M	65	174	106	43	Motorcycle	BiOM	87	56.3
14	M	28	175	86	6	Combat	WillowWood Pathfinder	86	62.5
15	M	65	175	98	16+	Combat	Ottobock Triton LP	93	64.5
16	M	67	175	83	16+	Combat	Freedom Sierra	96	62.5
17	M	64	177	80	13	Motorcycle	Harmony Triton	84	62.5
18	M	64	178	99	1	Vascular	Freedom Thrive	86	54.4
**Mean** **(SD)**		**58** **14**	**176** **6**	**89** **14**	**13** **12**			**82** **12**	**55.8** **5.9**

TSA = Time Since Amputation; ABC = Activities-Specific Balance Confidence; PLUS-M = Prosthetic Limb Users Survey of Mobility

### Static stability

**Fig 5:**
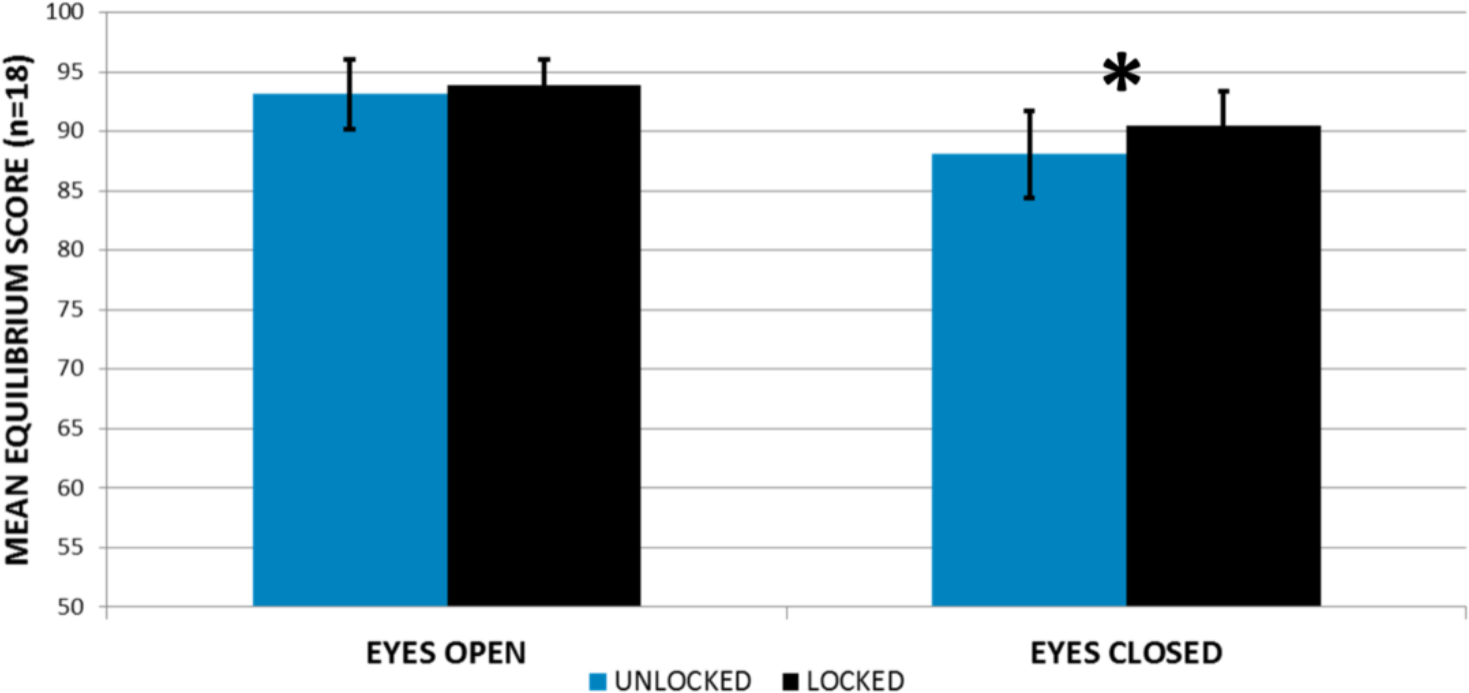
Mean (±SD) equilibrium scores for quiet standing balance tests (eyes open and eyes closed) with the bimodal ankle-foot system unlocked (curved effective rocker shape) and locked (flat effective rocker shape). An asterisk denotes a statistically significant difference (*p*<0.05).

**Fig 6:**
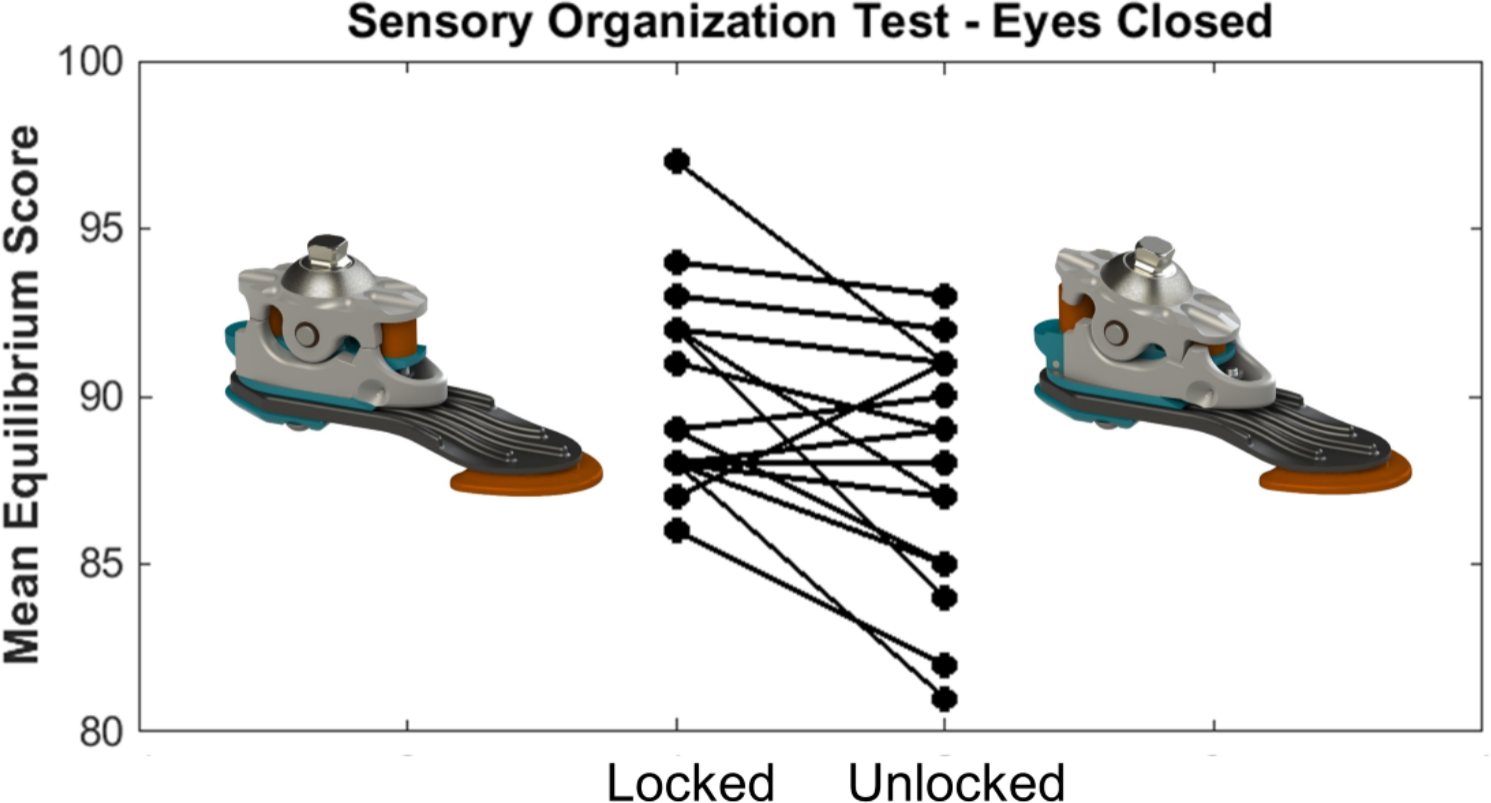
Mean equilibrium score for individual subjects standing with their eyes closed while using the locked and the unlocked bimodal ankle-foot system.

Mean SOT results are shown in Fig 5 for both the LOCKED and UNLOCKED conditions. Statistical analysis revealed a significant interaction term between MODE and EYES (F(1,17) = 6.759, *p* = 0.019), in which subjects exhibited a higher equilibrium score when standing on the LOCKED (90.5 ± 2.8) versus the UNLOCKED (88.1 ± 3.6) ankle with their eyes CLOSED. A post-hoc analysis indicated that this difference was statistically significant (*p* = 0.004) and present in 14 of 18 subjects (Fig 6). No statistical differences were noted between the LOCKED (93.9 ± 2.1) and the UNLOCKED (93.1 ± 2.9) ankle when subjects stood with their eyes OPEN (*p* = 0.097).

### Dynamic stability

**Fig 7:**
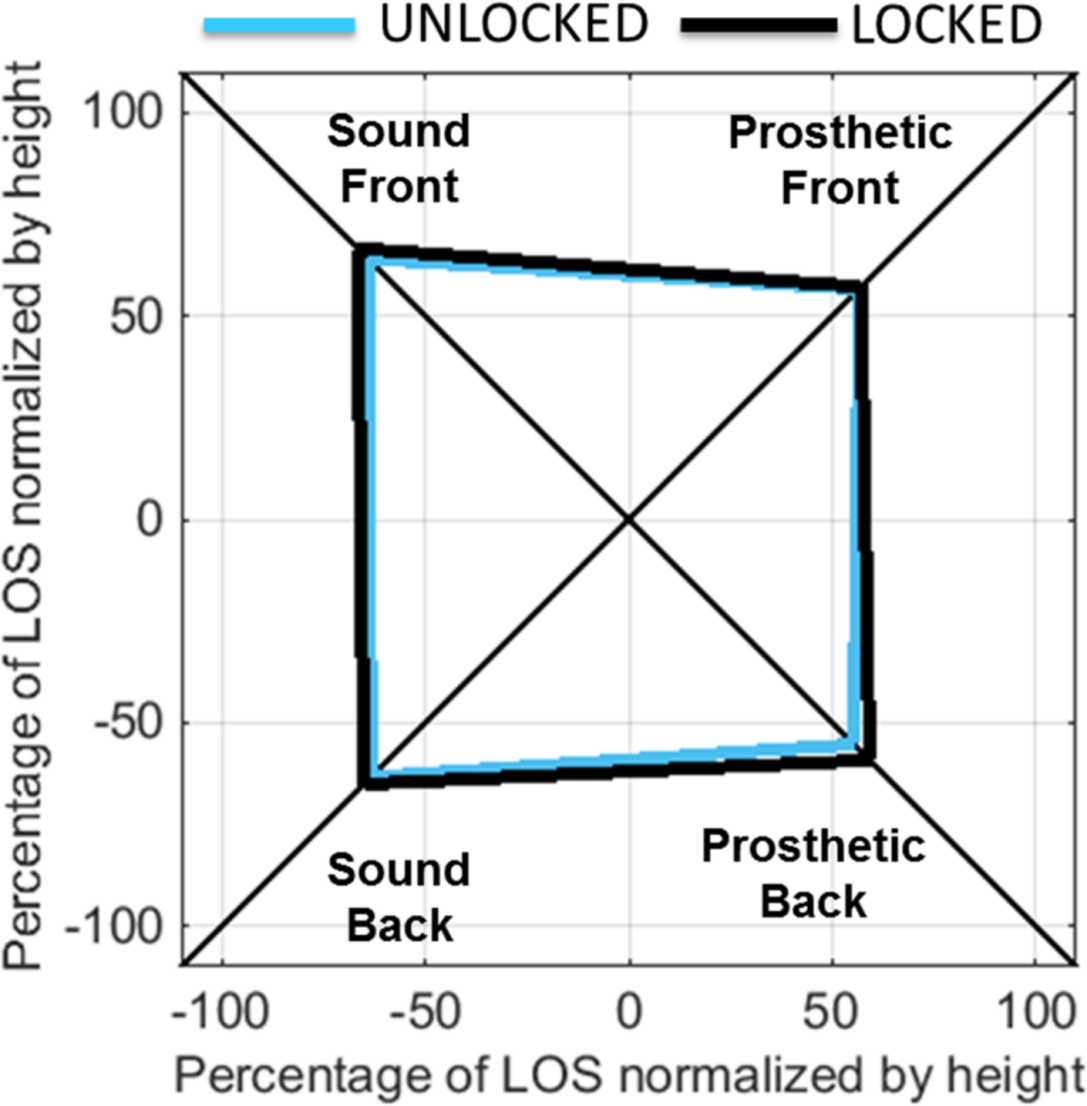
Mean maximum center of mass (COM) excursion, shown as a percentage of Limits of Stability (LOS) normalized by body height, when subjects used the unlocked (blue) and locked (black) bimodal ankle-foot system.

Mean LOS results are shown in Fig 7 for both the LOCKED and UNLOCKED conditions. The mean maximum COM excursion toward target 1 (expressed as a percentage of LOS normalized by height) was 80.8 ± 20.2% for LOCKED and 80.1 ± 23.1% for UNLOCKED; the mean maximum COM excursion toward target 2 was 83.7 ± 18.4% for LOCKED and 78.3 ± 21.6% for UNLOCKED; the mean maximum COM excursion toward target 3 was 92.1 ± 16.7% for LOCKED and 89.6 ± 15% for UNLOCKED; and the mean maximum COM excursion toward target 4 was 94.2 ± 19.1% for LOCKED and 90.6 ± 21.4% for UNLOCKED. Overall, subjects exhibited a reduced base of support toward their prosthetic side compared to their sound side for both the forefoot and heel regions. With the ankle in the LOCKED mode, 8 of 18 subjects were able to increase their maximum COM excursion toward the prosthetic forefoot and 11 of 18 subjects were able to increase their maximum COM excursion toward their prosthetic heel. However, statistical analysis did not reveal a significant main effect of MODE (F(1,17) = 3.593, *p* = 0.075) or a significant interaction term between MODE and TARGET (F(3,51) = 0.565, *p* = 0.641) across subjects. In an effort to compare subjects who improved their maximum COM excursion toward the prosthetic heel region when the ankle was LOCKED versus those who did not, an independent t-test was performed on a variety of demographic factors including age, body mass, height, ABC, and PLUS-M scores. With the exception of body mass, no significant differences were found between subjects who increased their COM excursion toward their prosthetic heel when the ankle was LOCKED compared to those who did not (two-tail *p*-values assuming equal variances for age = 0.775, mass = 0.036, height = 0.848, ABC = 0.482, PLUS-M = 0.169). With respect to body mass, subjects who increased their COM excursion toward their heel during the LOCKED condition had a significantly higher body mass (94 ± 13 kg) compared to those who did not (81 ± 11 kg).

### Functional stability

Overall, results from the modified MCT test were unremarkable given that most subjects did not experience a balance failure when the ankle was LOCKED or UNLOCKED regardless of perturbation direction or magnitude. In fact, only two subjects experienced a single balance failure (i.e., they grabbed the visual surround of the NeuroCom), one during a LOCKED condition and one during an UNLOCKED condition. This result represents two balance failures out of 648 total trials (2 perturbation directions x 3 perturbation magnitudes x 3 repetitions x 2 subject configurations on the force platform x 18 subjects).

### Mobility

Statistical analysis revealed no difference in the mean freely-selected walking speed (*p* = 0.08) of subjects walking with the UNLOCKED bimodal ankle (1.23 ± 0.22 m/s) compared to their usual prosthetic ankle-foot system (1.21 ± 0.21 m/s). Likewise, there were no statistically significant differences in mean L-test times (*p* = 0.5) when subjects walked with the UNLOCKED bimodal ankle (22.7 ± 4.5 sec) compared to their usual prosthesis (22.5 ± 5.1 sec).

### Qualitative feedback

Overall, 13 of 18 subjects liked the locking feature of the bimodal ankle and 14 of 18 subjects expressed interest in trying the system for a longer period of time in the future. When asked if they felt more balanced, less balanced, or the same during standing tasks when using the LOCKED ankle versus the UNLOCKED ankle, 10 of 18 subjects responded that they felt the same and 8 of 18 subjects responded that they felt more balanced with the LOCKED ankle. Of the 13 subjects who liked the locking feature of the ankle-foot system, 10 commented that this feature would be useful for activities that involved standing relatively still. Some of the activities mentioned were standing at a bar, standing on a boat, standing during archery or shooting, working with both hands at a counter, or reaching for an object. One of the subjects thought that the locking feature could be useful to brace himself when his young child ran up to him. Others thought that the locking feature would be useful for situations that involved movement, including skating, rock climbing, and for climbing a ladder.

Those who did not like the locking feature fell into two groups: those who had very low activity levels and those who had very high activity levels. The subjects with low activity levels felt that the feature was not necessary for them, as their activities were limited and did not require this adaptive feature. Those with very high activity levels were concerned that the ankle-foot system would not be able to respond quickly enough to their changing needs in each high movement situation and could be a hindrance in that situation.

To illustrate this concern, one subject, who worked as a first responder, indicated that during an emergency response one must have confidence that the foot can be used quickly (that it would be able to reach the unlocked state on the first step). Another subject (who was middle-aged) felt that someone older might benefit from the stability of the locked position.

Comments from participants in the non-structured interview portion of the study centered on a single important theme: control of the locking mechanism of the foot. Most subjects (15 of 18) indicated that in a future ankle-foot design, they would be interested in a system that could automatically lock when they were standing and automatically unlock when they were walking. Of these subjects, 8 of 15 indicated that they would like to manually override the automatic control feature of the ankle using a button on the prostheses, 6 of 15 indicated that they would like to use a key fob, and 1 of 15 indicated that they would like to use a mobile app.

## Discussion

Previous work suggests that two distinct functional modes are needed for ankle-foot prostheses to restore both effective walking mobility and stable standing balance [5]. To address the demands of standing and walking in individuals with lower-limb amputation, our group designed a novel bimodal ankle-foot prosthesis that can accommodate both functional modes using a rigid foot plate and an ankle that can lock (resulting in a flat effective rocker shape for standing) and unlock (resulting in a curved effective rocker shape for walking). The goal of this study was to examine the effect of the bimodal ankle-foot system on balance and mobility in a group of Veterans with unilateral transtibial amputation. Overall, we expected that subjects would exhibit an improvement in static, dynamic, and functional balance when using the bimodal ankle in the locked mode compared to the unlocked mode and that the unlocked bimodal ankle would perform similarly to the user’s usual prosthetic ankle-foot system during functional walking tasks.

Computerized dynamic posturography (CDP) was used in this study to objectively quantify static, dynamic, and functional balance. Compared to clinical outcome measures that are often used to evaluate the overall balance performance of individuals with lower-limb amputation (e.g., Timed “Up & Go” Test [22], Amputee Mobility Predictor [23], Two-Minute Walk Test [24], and the Functional Independence Measure [25]), CDP has the ability to objectively isolate and quantify different aspects of balance impairments. Among the various balance-measuring protocols administered within CDP, the SOT identifies abnormalities in an individual’s use of three sensory systems that contribute to static postural stability (i.e., visual, vestibular, and somatosensory). The SOT has been used in studies of lower-limb amputees to determine the effect of prosthetic components on balance [26], to identify amputees who are prone to falling [27], and to compare the balance performance of individuals with vascular versus traumatic amputation [28]. Another protocol administered within CDP is the LOS test, which assesses the ability of individuals to volitionally perturb their balance to explore their LOS. The LOS test has been used in studies of lower-limb amputees to assess the dynamic postural stability of this population compared to an able-bodied population [29]. It has also been used to study the effect of prosthetic alignment [30]. Most notably, Barnett et al. [19] used the LOS test (with 8 directions of leaning) to show significant reductions in the endpoint excursion of the COM on the prosthetic side compared to the sound side of unilateral transtibial amputees. Barnett et al. [19] also found that individuals with amputation expand their LOS during the first months after their discharge from rehabilitation, with no changes in the endpoint excursion of the COM between three and six months. Finally, the MCT, which quantifies functional postural stability, assesses the ability of an individual to recover from an unexpected postural disturbance. To date, few studies have used the MCT to evaluate functional postural stability among lower-limb amputees [27].

With regard to the use of these three balance metrics, results from the current study indicate that several domains of balance were improved when subjects stood with the bimodal ankle in the locked mode. Most notably, during the eyes closed condition of the SOT, subjects exhibited a significantly higher equilibrium score when standing with the locked ankle compared to the unlocked ankle. This result suggests that in the absence of visual feedback, the locked bimodal ankle-foot system may improve static balance for a subset of experienced, relatively active lower-limb prosthesis users. However, given the modest increase in equilibrium scores between the unlocked and locked conditions (2.4 ± 3.1), it is unclear whether this improvement represents a clinically meaningful change in the ability of users to maintain static balance throughout their daily lives. Previous studies of transtibial prosthesis users have found the average eyes closed SOT equilibrium score to fluctuate by as little as 1.6 points during a two-week, test-retest reliability study [31] and by as much as 7.8 points during a five-month longitudinal study [19]. To determine the significance of the results reported in the current study, future testing should focus on new amputees and lower mobility users, whose balance may be disproportionately impaired and for whom the bimodal ankle may provide an even greater benefit. In addition, given the potential for the bimodal ankle to improve static balance in situations when other contributors to balance are compromised, amputees with diabetes mellitus (especially those whose vision may be impaired due to diabetic retinopathy) and vestibular deficits (i.e., those whose amputation was cause by blast injuries or other traumatic injuries) may find the bimodal ankle to be particularly beneficial.

With regard to dynamic balance, subjects in this study consistently favored their sound side during LOS testing such that their functional base of support was smaller toward their prosthetic forefoot and their prosthetic heel. This finding is consistent with the results of previous studies, highlighting the key role of the sound side in maintaining balance following amputation [19,20]. Contrary to our hypothesis, subjects did not consistently increase their functional base of support toward their prosthetic side when the bimodal ankle was locked, as indicated by a relatively similar average base of support region between the locked and unlocked conditions shown in Fig 7. However, trends were observed toward the prosthetic heel region for 11 of 18 subjects, who increased their maximum COM excursion toward their prosthetic heel when the bimodal ankle was locked. This increase in functional base of support toward the heel region is logical, especially when considering that many prosthetic ankle-foot systems have soft heel regions for shock absorption in the early stance phase of walking. Accordingly, the ability of the bimodal ankle to lock and provide a flat heel region may be particularly effective at improving stability toward the posterior region of the user’s functional base of support.

In this study, very few balance failures were caused by perturbations to the standing surface during functional balance testing, regardless of whether the bimodal ankle was locked or unlocked. Several factors are likely related to this outcome, including the possibility that the perturbation protocol implemented in this study was not aggressive enough to destabilize the subject even when modifications to the MCT protocol were implemented to specifically challenge balance toward the prosthetic forefoot and heel regions. In addition, subjects were able to focus their attention on the perturbation task in this study without an external cognitive load, which may have decreased the difficulty of the task and allowed subjects to more easily recover from balance perturbations. Finally, as discussed with other testing results, many of the subjects in this study had high balance confidence and self-perceived mobility ratings, suggesting that their ability to recover from a balance perturbation was probably above average for a lower-limb prosthesis user. Although the inclusion criteria used in this study did not necessarily target higher mobility users, subjects who are more mobile are probably more likely to volunteer for research studies that require travel outside of the home, resulting in an unbalanced sample population. Consequently, the results described in this study do not adequately address the performance of lower mobility users for whom the bimodal ankle may provide the most benefit (e.g., Medicare Functional Classification Level K1 and K2 prosthesis users). To selectively recruit a population of lower mobility users, future studies should consider testing the effect of the bimodal ankle in a home setting (i.e., outside of a laboratory or hospital facility), which will likely encourage the involvement of subjects who may otherwise be unable to travel outside of their home for a research study.

As expected, subjects had similar walking speeds and L-test times when using the unlocked bimodal ankle compared to their usual ankle-foot prosthesis, confirming that the unlocked bimodal ankle did not compromise mobility compared to the subjects’ usual prosthetic ankle-foot system. In addition to endorsing the use of the bimodal ankle for level walking, this finding highlights the fact that rigid-keel flexible ankle designs have the potential to provide mobility outcomes similar to flexible keel systems. Future work, however, should focus on characterizing the rotational impedance of elastic elements that control the ankle range-of-motion of the bimodal ankle so that the walking mode of the system can be optimized to meet the functional demands of the user.

In addition, future studies of the bimodal ankle-foot system should investigate the long-term effect of using the ankle during activities of daily living, which would allow users to more fully acclimate to the features of the system. Future studies should also evaluate the extent to which users compensate for static, dynamic, and functional balance requirements by relying on kinematic strategies on their sound side and at joints proximal to their amputation. Unfortunately, kinematic data were not collected in this study, making it difficult to attribute improvements in balance performance to the design features of the bimodal ankle alone. Alternatively, kinematic adaptations at joints proximal to the amputation, such as increased knee flexion or trunk lean, may have simultaneously contributed to balance improvements, exaggerating the assumed effect of the bimodal ankle, or in some cases, compensating for ankle motion permitted during the unlocked condition such that minimal differences were observed between the locked and unlocked conditions. It is also possible that the rigid foot plate of the bimodal ankle may have improved balance measures in the coronal plane, which were not investigated in this study.

Overall, the results of this study support further research and development of the bimodal ankle-foot prosthesis. To enhance the versatility of this system, it may be worthwhile to implement a design feature that would allow the ankle to lock at different angles, which could improve balance function during common activities of daily living (e.g., slope standing). In addition, qualitative comments collected during this study suggest that prior to commercial implementation, future design iterations should incorporate: 1) an automatic switching mode between the locked and unlocked conditions so that users can effortlessly transition between standing and walking and 2) physical controls on the prosthesis or a portable key fob to allow for the manual override of the automatic mode. Recent efforts to develop a fast and accurate automatic switching mode using machine learning algorithms as well as easy-to-use physical controls on the prosthesis appear promising [32]. Accordingly, further design enhancements in this direction are likely to improve technology transfer efforts such that a bimodal ankle-foot system may one day be commercially available to individuals with lower-limb amputation.

## Supporting information

S1 File Minimal data underlying study results. (XLSX)
